# What is the role of institutional quality in health outcomes? A panel data analysis on 158 countries from 2001-2020

**DOI:** 10.1016/j.heliyon.2023.e20251

**Published:** 2023-09-17

**Authors:** Alireza Hadipour, Sajad Delavari, Mohsen Bayati

**Affiliations:** a Student Research Committee, School of Health Management and Information Sciences, Shiraz University of Medical Sciences, Shiraz, Iran; b Health Human Resources Research Center, School of Health Management and Information Sciences, Shiraz University of Medical Sciences, Shiraz, Iran

**Keywords:** Life expectancy, Infant mortality rate, Control of corruption, Government effectiveness, Rule of law, Regulatory quality, Institutional quality

## Abstract

Our study investigated the impact of institutional quality on health system outcomes, utilizing worldwide governance indicators and analyzing data from 158 countries between 2001 and 2020. We employed Principal Component Analysis (PCA) to create a composite index of institutional quality and conducted various tests to select the appropriate econometric model. The role of institutional quality, along with other variables, in health outcomes was estimated using fixed effects and generalized method of moments (GMM) models. High-income and low-income countries were analyzed separately. The results of our study revealed that institutional quality, as measured by Control of Corruption, Voice and Accountability, Political Stability, Rule of Law, Regulatory Quality, and Government Effectiveness, had a negative impact on infant mortality rates and a positive impact on life expectancy. Similarly, variables such as GDP, mean years of schooling, total health expenditure, and urbanization rate showed a negative association with infant mortality rates and a positive association with life expectancy. Conversely, the logarithm of CO2 emissions exhibited a positive effect on infant mortality rates and a negative effect on life expectancy. These findings highlight the crucial role of institutional quality in determining health outcomes. Improving institutional quality contributes to the development of democratic and meritocratic systems, infrastructure enhancement, efficient tax and subsidy systems, optimal budget allocation, improved public education, and enhanced access to primary healthcare services. The influence of institutional quality is particularly significant in high-income countries compared to low-income countries. In conclusion, our study emphasizes the importance of institutional quality in shaping health system outcomes. Enhancing institutional quality is essential for the overall advancement of healthcare systems, encompassing governance, infrastructure, education, and access to healthcare services. It is crucial to prioritize efforts to improve institutional quality, especially in high-income countries, to achieve better health outcomes for populations worldwide.

## Graphical abstract

**Figure:**
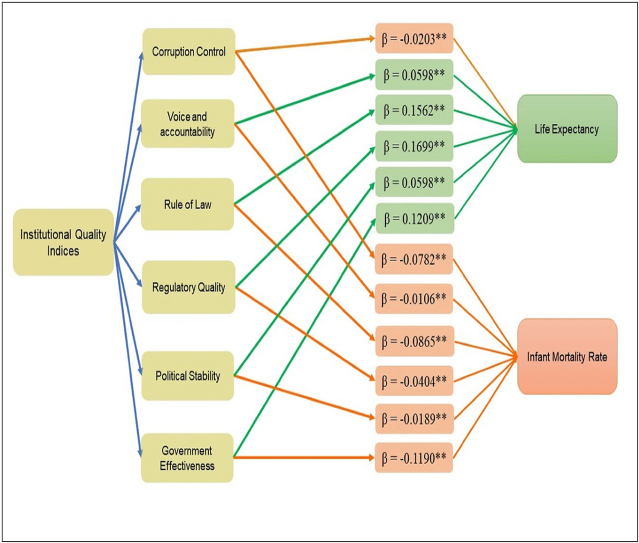

## 1 Introduction

The future presents societies with a wide array of crucial challenges, including climate change, the emergence of new viruses and epidemics, wars and terrorism, economic crises, high living costs, and healthcare system crises. However, at the heart of these challenges lies a fundamental crisis in governance [1]. In essence, the fundamental institution in society responsible for addressing the mentioned challenges is the governance. Additionally, to effectively confront these challenges, the governance must establish specialized systems tailored to address them in a targeted manner [1,2]. Thus, the governance assumes the role of designing and implementing the health system to address the society's health-related challenges [3].

Accordingly, and based on 2000 report published by World Health Organization (WHO), the governance is expected to design the country's health system with four primary functions: provision, production of resources, service provision, and financing to achieve three overarching goals: responsiveness to non-medical expectations of individuals, fair financial contributions, and overall enhancement of public health [3,4].

**Fig. 1:**
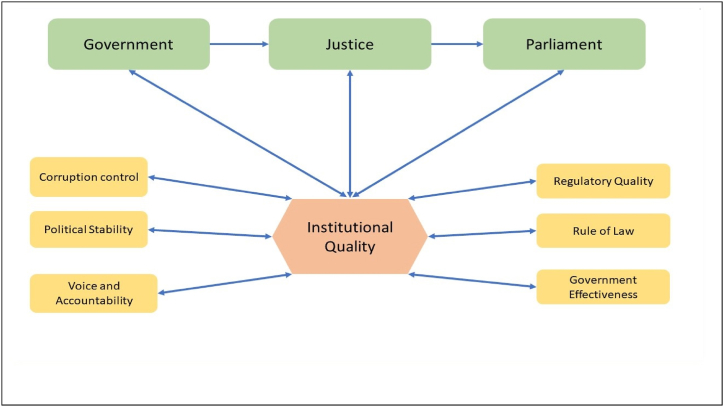
Institutional quality along with its constituent components and the corresponding evaluation indicators.

Governance, as defined by the World Bank, encompasses the traditions and institutions that hold authority within a country. This broad definition includes the processes of selecting, monitoring, and replacing governments [5]. In the realm of economic literature, governance refers to the collective institutions comprising the government, judiciary, and parliament [6,7]. Daniel Kaufmann et al. (1999) introduced six indicators, namely Control of Corruption, Voice and Accountability, Political Stability and Absence of Violence/Terrorism, Rule of Law, Regulatory Quality, and Government Effectiveness, to assess institutional quality [8]. Fig. 1 illustrates institutional quality along with its constituent components and the corresponding evaluation indicators.

Unfortunately, there is a limited body of research investigating the role of institutional quality and its sub-indices in the healthcare sector. For instance, Socoliuc et al. (2022) demonstrated that within the European Union, higher institutional quality is associated with reduced child mortality rates and improved life expectancy [9]. Similarly, Rahman et al. (2022) indicated that good governance, economic growth, and renewable energy consumption contribute to improved life expectancy in ANZUS-BENELUX countries [10]. Moreover, Vian (2020) emphasized that corruption undermines the capacity of health systems to promote better health, economic growth, and overall development. Therefore, addressing corruption through interventions and allocating resources towards prevention and control are crucial components of strengthening health systems for Universal Health Coverage (UHC) [11]. Additionally, Koller et al. (2020) demonstrated that anti-corruption policies, transparency, and accountability lead to enhanced access to physicians, nurses, medical drugs, and the development of UHC [12]. Furthermore, Achim et al. (2020) revealed that corruption has a negative impact on life expectancy and happiness [13]. Mackenbach and McKee (2015) found a significant positive association between the quality of democracy, quality of government, and process and outcome indicators of healthy policies [14]. Similarly, Makuta and Bernadette (2015) demonstrated that better governance quality is associated with improved health outcomes in Sub-Saharan Africa, indicating that public health expenditure is more effective in countries with higher levels of governance quality compared to those with lower levels [15].

Regrettably, there has been a lack of comprehensive research exploring the relationship between institutional quality and its impact on health outcomes, resulting in significant knowledge gaps. Existing studies have primarily focused on examining the link between democracy indicators and health outcomes, such as infant mortality or life expectancy [14]. However, the role of institutional quality has received limited attention.

While studying the impact of democracy indicators on health system performance is valuable, it is essential to delve deeper and investigate the influence of the quality of institutions that underpin these democracy indicators. Therefore, the objective of this study is to bridge these gaps by examining the role of institutional quality and its sub-indices in relation to life expectancy and infant mortality rates, serving as crucial measures of health system performance.

In the following, we will begin by introducing the theoretical framework and conducting a review of relevant studies, with a particular focus on explaining two hypotheses. Subsequently, we will proceed to describe the variables and econometric methods employed in the analysis. Finally, we will elaborate on the obtained results and thoroughly discuss their implications.

## 2 Literature review and hypothesis development

The notion of institutional quality in economics boasts a rich historical background, marked by early recognition of institutions' profound influence on economic outcomes and societal advancement. The formal emergence of institutional economics as a distinctive field took place in the latter half of the 20th century, delving into the intricate relationship between institutions and economic development [43]. In the mid-20th century, economists turned their attention to scrutinizing the impact of legal systems, property rights, and governance structures on economic growth, with Douglass North's contributions greatly shaping this domain. The exploration of institutional quality further expanded to encompass the study of inclusive institutions that foster economic opportunity and innovation, while simultaneously identifying extractive institutions as impediments to both growth and equity [44,45]. In more recent times, the investigation into institutional quality has evolved, leveraging advanced econometric techniques and enhanced data accessibility, thereby accentuating the pivotal role of robust governance and sound institutions in tackling global challenges. The historical trajectory of institutional quality underscores the indispensable role institutions play in advancing economic prosperity and societal well-being, thus elevating institutional economics as a crucial research avenue for addressing the intricacies of the contemporary world [[46], [47], [48], [49], [50]].

In addition, the World Health Organization (WHO) emphasized governance as the primary function of health systems in countries, highlighting the significance of governance and its role within national health systems. Furthermore, the WHO consistently acknowledges the crucial and profound role of governance ND it's quality in healthcare systems, discussing its impact on healthcare goals and outcomes [[4], [5], [6], [7], [8], [9], [10], [11], [12], [13], [14], [15], [16], [17], [18], [19], [20], [21], [22], [23], [24], [25], [26], [27], [28], [29], [30], [31], [32], [33], [34], [35], [36], [37], [38], [39], [40], [41], [42], [43], [44], [45], [46]].

Accordingly, this study investigates two main hypotheses.

1. Improving institutional quality indicators has led to a decline in infant mortality rates across countries globally from 2001 to 2020.
2. Improving institutional quality indicators has resulted in an increase in life expectancy across countries globally from 2001 to 2020.

We firmly believe that the enhancement of institutional quality indicators, such as Control of Corruption, Voice and Accountability, Political Stability and Absence of Violence/Terrorism, Rule of Law, Regulatory Quality, and Government Effectiveness, has resulted in a reduction in infant mortality rates (first hypothesis) and an increase in life expectancy levels (second hypothesis). This effect holds true for both high and low-income countries, indicating that improved governance positively impacts the healthcare achievements of nations across different income groups. In essence, governance plays a vital and influential role in the health systems of countries. By examining its impact on healthcare outcomes through a global, long-term, and comprehensive approach, we can effectively enhance the performance of healthcare systems and achieve the fundamental goal of promoting and maintaining the health of society. Understanding the significance of governance in healthcare is crucial for addressing the complexities of health challenges and advancing the well-being of populations worldwide.

Unfortunately, despite the significant importance of investigating the role of institutional quality in the performance and objectives of healthcare systems in countries, there is a scarcity of studies in this area that explore certain indicators, such as corruption control, government effectiveness, democracy etc. For instance, in a study conducted by Socoliuc et al. (2022), the impact of anti-corruption policies on life expectancy and child mortality was examined. The researchers utilized data from European Union countries spanning the years 2000–2019 and employed a panel data approach for analysis. The findings of the study revealed that the enhancement of anti-corruption policies across various sectors, including the health sector, has resulted in a notable reduction in infant mortality rates and an overall improvement in life expectancy within EU countries. These results emphasize the importance of anti-corruption measures in promoting positive health outcomes and longevity in the context of a nation's healthcare system [9], also, Cárcaba et al. (2022) investigated the effects of good management in local governments on individual subjective well-being in Spain during the period from 2013 to 2018. The study defined three dimensions of good governance at the municipal level, namely accountability, government efficiency, and control of corruption. The results of our study reveal that with respect to good governance, there is an immediate positive influence of government efficiency on individual subjective well-being (SWB) levels. On the other hand, transparency, which represents accountability, does not seem to have a significant effect. Interestingly, we did not observe an immediate impact of corruption on reported SWB, but we found a substantial delayed effect [40]. Moreover, Abbas et al. (2023) investigated the factors affecting administrative state capacity in Asia, particularly bureaucratic quality (BQ) and military involvement in politics (MP), and their impact on sustainable public health quality (PHQ) from 2006 to 2020. It focuses on the implementation of Goal 04: Health and Well-being within the Sustainable Development Goals, taking into account the administrative challenges in Asian countries. The research reveals that insufficient BQ, influenced by political pressures and policy inconsistencies, has significantly and negatively affected PHQ in Asia. Conversely, a strong presence of MP and military influence has positively influenced PHQ in the region. However, the interaction between BQ and MP has presented challenges for the state's social development, arising from conflicting interests and policy outcomes [47]. Additionally, Rosenberg (2018) demonstrates that good governance contributes to a reduction in infant mortality rates, and democracy can further strengthen this effect. Therefore, Studies examining the impact of institutional quality indicators on health system outcomes have consistently demonstrated the positive and significant role of improving institutional quality in enhancing health system performance. However, it is unfortunate that the number of such studies remains very limited [17]. Finally, Miao et al. (2023) In this study, Asian panel analysis was employed to assess institutional quality. The findings demonstrated that a corruption-free environment, political stability, and accountability are the most influential factors in determining institutional quality, which in turn, positively impacts the economy's performance and growth. The study delved deeply into governance practices, particularly focusing on developing economies with low Human Development Indices and facing overpopulation crises. It was observed that state fragility, characterized by weak social, economic, and political cohesion, negatively affects the Composite Index of Institutional Quality (CPIAQ). Furthermore, the study highlighted the adverse effects of external intervention from political, economic, social, and regional forces. Such interventions impose pressures on state institutions, leading to the implementation of standardized regulations without considering the institutions' capacity and available resources. This hampers institutional quality and undermines the effectiveness of state institutions [50].

Furthermore, various studies have investigated the impact of additional indicators on the health outcomes of countries. These indicators include gross domestic product (GDP), the proportion of total health costs in national income, education level, urbanization rate, and greenhouse gas emissions such as carbon dioxide. For instance, Roffia et al. (2023) explored the determinants of life expectancy in OECD countries and found that GDP per capita, health expenditure, bed, and physician density have a positive effect on life expectancy, while the prevalence of chronic diseases and temperature have a negative effect [48]. Moreover, Udemba et al. (2023) examined the environmental implications of energy policies and private and public subsidies on infant mortality rate in India. Their results indicate that renewable energy sources and social factors (GDP per capita), along with public subsidies (general government final consumption expenditure), have a mitigating impact on infant mortality in India. Conversely, private subsidies (gross capital formation), fossil fuels, and carbon dioxide contribute to an increase in infant mortality rates in the country [49]. In addition, Rahman and Alam (2022) demonstrated that renewable energy, economic growth, good governance, and urbanization have a positive effect on life expectancy. For every 1% increase in these variables, life expectancy increases by 0.009%, 0.070%, 0.022%, and 0.107%, respectively. On the other hand, environmental pollution negatively impacts life expectancy, with an elasticity of −0.015 [10].

Therefore, it can be asserted that various studies, including those mentioned in this section, have consistently demonstrated the positive and significant impact of various factors, such as institutional quality, democracy, gross domestic product, improved health expanditure, and higher education levels, in enhancing life expectancy and reducing infant mortality. However, two primary criticisms exist.

First, one of the main criticisms is the limited scope of the studies, which often focus solely on indicators like corruption control and government effectiveness while neglecting other crucial indicators, such as the voice and accountability, the rule of law, and the quality of laws and regulations.

Secondly, there is a notable lack of research that adopts a comparative approach, analyzing the role of institutional quality indicators and other factors influencing health outcomes across different income groups. Additionally, the absence of a comprehensive global approach in these studies has hindered a holistic understanding of the relationship between institutional quality and health outcomes worldwide.

Accordingly, our study aims to bridge the gap in existing research and explore the role of institutional quality indicators and other socio-economic determinants in shaping life expectancy and infant mortality rates across countries of the world, categorized by income groups.

The acceptance or rejection of the aforementioned research hypotheses is given in the results table (Table 3, Table 4) of the models.

## 3 Materials and methods

**Fig. 2:**
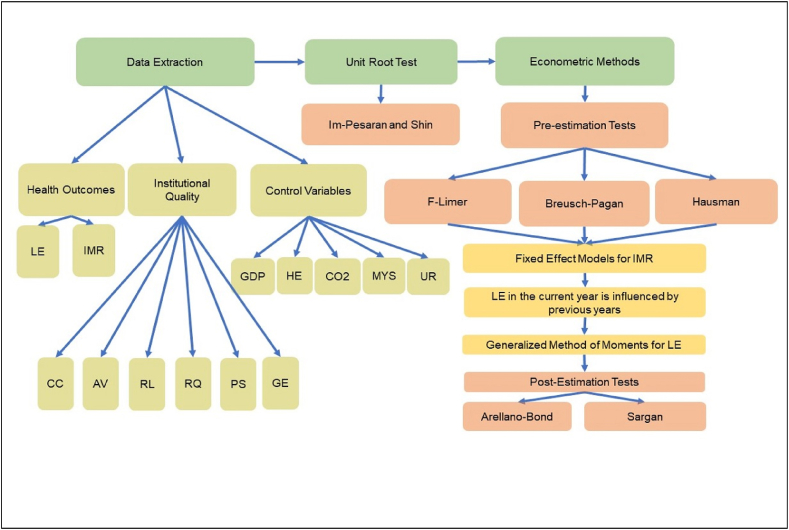
The steps of data extraction to choose econometric model.

According to Fig. 2, the initial step involved extracting data pertaining to health outcomes, institutional quality, and control variables from international databases. Subsequently, the presence of a unit root was examined using the Im-Pesaran and Shin test. The subsequent stage encompassed econometric modeling, wherein the estimator type was determined through pre-estimation tests. Lastly, post-estimation tests were conducted to assess the precision of the outcomes.

### 3.1 Data

We conducted our analysis using a sample of 158 countries worldwide, spanning the period from 2001 to 2020 (N = 3160). The selection of these countries was based on the literature and availability of data for the entire duration. To ensure the reliability of our findings, we sourced the data from reputable international organizations such as the World Health Organization (WHO), the World Bank (WB), and the United Nations (UN) databases.

Our study aimed to assess the impact of institutional quality on health outcomes, with life expectancy (LE) and the logarithm of infant mortality rate per 100,000 (LIMR) as the dependent variables. We considered Institutional Quality (IQ) as the primary explanatory variable of interest. Furthermore, drawing from existing literature [13,16,17], we incorporated several control variables in our analysis. These control or moderating variables included Total Health Expenditure as a percentage of GDP (HE), the logarithm of real Gross Domestic Production (PPP) (LGDP), Mean Years of Schooling (MYS), Urbanization Rate as a percentage of the total population (UR), and the logarithm of CO2 emissions (LCO2).

To assess institutional quality (IQ), we utilize various sub-indices obtained from the Worldwide Governance Indicators (WGI) developed by the World Bank. The sub-indices we employ include the following: Control of corruption (CC), Voice and Accountability (VA), political stability and absence of violence/terrorism (PS), Rule of Law (RL), Regulatory Quality (RQ), and Government Effectiveness (GE). In order to construct a composite index for IQ, we combine these sub-indices using a methodology similar to Khan et al. [7] and Eslamloueyan et al. [6], which involves employing Principal Component Analysis (PCA). By applying PCA, we can effectively merge the IQ sub-indices into a single composite index. It is worth noting that both the IQ composite index and its sub-indices variables range from −2.5 to +2.5. The data variables, their measurements and resources are described in Table 1.

**Table 1:** Data, variables and measurements.

Variables	Type	Description	Scale	Reference	Source
LE (Years)	Dep	The average number of years a person is expected to live, based on statistical data and population demographics.	0–inf	[[10], [11], [12], [13], [14], [15], [16], [17], [18], [19], [20], [21], [22], [23], [24], [25], [26], [27], [28], [29], [30], [31], [32], [33], [34], [35], [36], [37], [38], [39], [40], [41], [42], [43], [44], [45], [46], [47], [48]]	WHO + WB
IMR (Per 100,000)	Dep	Infant mortality rate (IMR) refers to the number of deaths of infants under one year of age per 1000 live births in each population during a specific time period.	0-inf	[[33], [34], [35], [36], [37], [38], [39], [40], [41], [42], [43], [44], [45], [46], [47], [48], [49]]	WHO + WB
IQ	Indep	The combined of 6 worldwide Governance indicators by PCA	−2.5/+2.5	[[14], [15], [16], [17], [18], [19]]	WB
CC	Indep	Control of corruption is a metric that gauges the perceptions of how much public power is wielded for personal gains, encompassing various forms of corruption, both minor and significant.	−2.5/+2.5	[[9], [10], [11], [12], [13]]	WB
VA	Indep	Voice and accountability capture perceptions of the extent to which a country's citizens are able to participate in selecting their government, as well as freedom of expression, freedom of association, and a free media.	−2.5/+2.5	[[9], [10], [11]]	WB
PS	Indep	Political Stability and absence of violence and terrorism measures perceptions of the likelihood of political instability and/or politically motivated violence, including terrorism.	−2.5/+2.5	[32,33]	WB
RL	Indep	Rule of law captures perceptions of the extent to which agents have confidence in and abide by the rules of society, and in particular the quality of contract enforcement, property rights, the police, and the courts, as well as the likelihood of crime and violence.	−2.5/+2.5	[[14], [15], [16], [17], [18], [19], [20], [21], [22], [23], [24], [25], [26], [27], [28], [29], [30], [31], [32], [33], [34], [35]]	WB
RQ	Indep	Regulatory Quality captures perceptions of the ability of the government to formulate and implement sound policies and regulations that permit and promote private sector development.	−2.5/+2.5	[[14], [15], [16], [17], [18], [19], [20], [21], [22], [23], [24], [25], [26], [27], [28], [29], [30], [31], [32], [33], [34], [35]]	WB
GE	Indep	Government Effectiveness captures perceptions of the quality of public services, the quality of the civil service and the degree of its independence from political pressures, the quality of policy formulation and implementation, and the credibility of the government's commitment to such policies.	−2.5/+2.5	[[37], [38], [39]]	WB
HE (%GDP)	Con	The total ratio of three indicators of public sector payments, patients' out-of-pocket payments and private sector payments in the health sector to GDP	0–100%	[[10], [11], [12], [13], [14], [15], [16], [17], [18], [19], [20], [21], [22], [23], [24], [25], [26], [27], [28], [29], [30], [31], [32], [33]]	WHO + WB
GDP (PPP)	Con	a fundamental economic indicator used to measure the total value of all goods and services produced within a country's borders during a specific time period, typically a year or a quarter.	0-inf	[[18], [19], [20], [21], [22], [23], [24], [25]]	WB
MYS (Years)	Con	It measures the average number of years of formal schooling that individuals aged 25 years and older have received, regardless of when they completed their education.	0-inf	[[10], [11], [12], [13], [14], [15], [16], [17], [18], [19], [20]]	UN
UR (%TP)	Con	That measures the level of urban growth and the shift of the population from rural to urban areas over time.	0–100%	[[10], [11], [12], [13], [14], [15], [16], [17], [18], [19], [20], [21], [22], [23], [24], [25], [26], [27], [28], [29], [30], [31], [32], [33]]	WB
CO_2_ (Ton)	Con	CO2 emissions occur through various human activities, primarily related to the burning of fossil fuels such as coal, oil, and natural gas for energy production, transportation, industrial processes, and other economic activities.	0-inf	[41,42]	WB

Dep: Dependent variables, Indep: independent variables, Con: control variables, WHO: World Health Organization, WB: The World bank, UN: United Nations, inf: Infinity.

### 3.2 Models

To select the most appropriate estimation method, we conducted panel data diagnostic tests. These tests included the F-Limer test, which helped determine whether the model should be pooled or implemented as a Fixed Effect (FE) model. We also used the Breusch-Pagan test to choose between a pooled or Random Effect Model (REM), and the Haussmann test to distinguish between fixed and random effect models [18]. Additionally, we utilized the Im-Pesaran and Shin (IPS) test to examine the presence of unit roots in the panel data. Based on the results of these diagnostic tests, we decided to employ the panel and FE model for our analysis, as shown in Table A3.

To examine the impact of IQ on LIMR (logarithm of infant mortality rate), similar to Socoliuc et al. (2022) [9] and Rosenberg (2018) [17] we developed a panel model and estimated it using the Fixed Effect (FE) approach. The model is specified as follows:

(1) LIMRit = β0 + β1IQit + β2HEit + β3MYSit + β4URit + β5LGDPit + β6LCO2it + νit

Here, i ranges from 1 to 158 representing countries, and t ranges from 1 to 20 representing years. In this model, β0 represents the constant term, β1 to β6 are the coefficients corresponding to the explanatory and control variables, and νit represents the error term.

Furthermore, considering the assumption that, similar to Socoliuc et al. (2022) [9] and Rahman et al. (2022) [10] we developed a dynamic panel model to examine the role of IQ in LE (life expectancy). The dynamic panel model is expressed as follows:

(2) LEit = β0 + β1LEit-1 + β2IQit + β3HEit + β4MYSit + β5URit + β6LGDPit + β7LCO2it + νit

Similar to the previous model, i ranges from 1 to 158, and t ranges from 1 to 20. In this dynamic model, β0 represents the constant term, β1 represents the coefficient of the lagged LE variable, and β2 to β7 correspond to the explanatory and control variables. The term νit represents the error term in the model.

Estimating the dynamic panel data model using ordinary least squares (OLS) or general least squares (GLS) can give rise to a concern known as dynamic panel bias. This issue arises due to the potential lack of independence between the lagged dependent variable in the model and the error term. While the within estimator (WI) can eliminate individual effects, it may introduce a correlation between the transformed lagged dependent variable and the transformed error term. As a result, the WI estimator may exhibit a downward bias. However, this problem can be mitigated as the number of individuals (N) in the dataset increases while the time period (T) remains fixed. With a larger N, the bias in the WI estimator tends to diminish [21].

Another important issue to consider is the potential presence of reverse causality between LE and IQ. On one hand, a poor institutional environment can have a negative impact on LE. On the other hand, low LE can also influence the quality of institutions. This causal relationship introduces the possibility of endogeneity, which can complicate the estimation process. In order to address these problems, we employ the generalized method of moments (GMM) to estimate our dynamic panel data model. The GMM estimator helps overcome the endogeneity issue and provides consistent estimates for Equation (2). Moreover, GMM is capable of accounting for heteroscedasticity and autocorrelation, enhancing the robustness of the estimation. By utilizing this method, we can select parameters in a way that minimizes the correlation between the disturbance term and a set of instruments, aiming for a close-to-zero correlation [21,22].

In better words, the fundamental assumption when estimating with GMM is the presence of heterogeneity and heteroscedasticity between variables. As a result, after estimating the equations using GMM, there is no need to verify heterogeneity and heteroscedasticity the. For this reason, we did not perform the mentioned tests. Accordingly, we utilize the system GMM technique, which is an instrumental variable approach. This method employs lagged levels as instruments for first-differenced variables. However, it is worth noting that previous studies by Arellano and Bover (1995) and Blundell and Bond (1998) have indicated that lagged levels may sometimes serve as weak instruments for first-differenced variables. To overcome this limitation, these researchers extended the GMM estimator by incorporating both lagged levels and lagged differences. As a result, many researchers prefer to use this expanded estimator, referred to as the system GMM, to address the issue of weak instruments [[18], [19], [20], [21], [22]].

To assess the validity of the instrument sets used in our estimations, we utilize the Sargan test (J-statistics). The Sargan test examines the null hypothesis that the instruments, as a group, are exogenous. In addition, we conduct the Arellano-Bond AR (1) and AR (2) autocorrelation tests on the residuals. The null hypothesis of the AR (1) test is no autocorrelation. These tests provide insights into the reliability of our instrumental variables and the presence of autocorrelation in the model residuals [21,22].

Moreover, in addition to the composite index of institutional quality (IQ), we estimate the aforementioned models (equations (1), (2))) separately for each of the six sub-indices of IQ, namely Control of Corruption (CC), Voice and Accountability (VA), Political Stability (PS), Rule of Law (RL), Regulatory Quality (RQ), and Government Effectiveness (GE).

To compare the impact of institutional quality (IQ) on health outcomes (LE and LIMR) across different income levels, we conduct separate estimations of the models for countries in high-income and low-income groups. The classification of countries is based on the World Bank's income categorization. Specifically, we combine high-income and upper-middle-income countries (HICs and UMICs) into the high-income group (HIG), which includes 91 countries. Likewise, we combine lower-middle-income and low-income countries (LMICs and LICs) into the low-income group (LIG), comprising 67 countries. This approach allows us to analyze the relationship between IQ and health outcomes within each income group.

## 4 Results

**Fig. 3:**
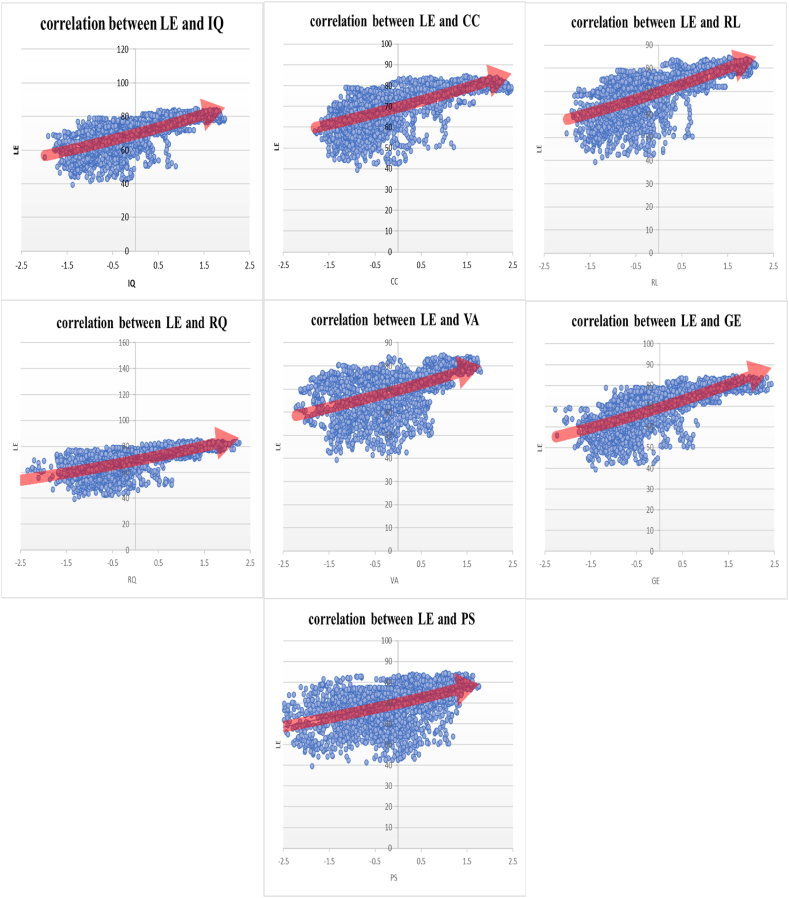
Correlation between life expectancy and institutional quality sub-indices (The vertical axis is the life expectancy of countries and the horizontal axis is the institutional quality sub-indices).

**Fig. 4:**
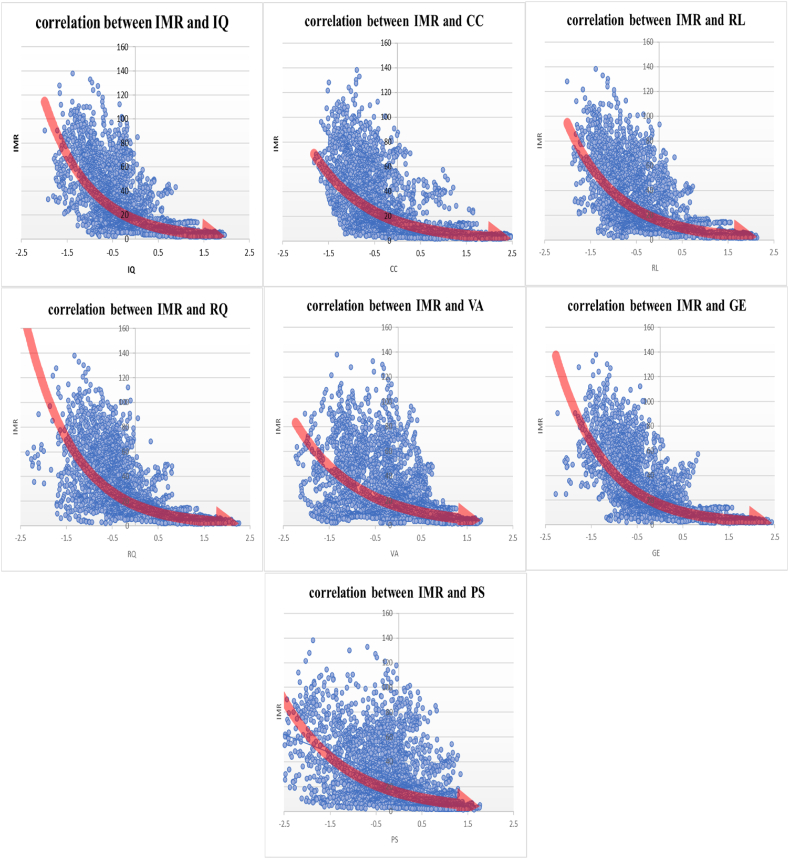
Correlation between infant mortality rate and institutional quality sub-indices (The vertical axis is the infant mortality rate of countries and the horizontal axis is the institutional quality sub-indices).

The correlation analysis conducted in this study demonstrates a positive correlation between institutional quality and life expectancy, as well as a negative correlation between institutional quality and infant mortality. Please refer to Fig. 3, Fig. 4 for a visual representation of these correlations.

Table 2 presents a comprehensive overview of the descriptive statistics for the variables examined in this study. The average life expectancy (LE) is 70 years, with Japan recording the highest LE and Sierra Leone having the lowest LE [4]. The mean infant mortality rate (LIMR) is 27 per 100,000 live births, with Sierra Leone experiencing the highest LIMR and Iceland having the lowest LIMR [4]. The average institutional quality (IQ) is −0.0299, and the sub-indices of control of corruption (CC), voice and accountability (VA), political stability (PS), rule of law (RL), regulatory quality (RQ), and government effectiveness (GE) have mean values of −0.0392, 0.0442, −0.1084, −0.0419, 0.0437, and 0.0173, respectively. Finland ranks highest in terms of IQ, while Afghanistan ranks lowest in this regard [23]. Furthermore, the average total health expenditure (HE) accounts for 6.1327% of GDP. The United States of America demonstrates the highest HE, while the Republic of Congo has the lowest, HE. The average gross domestic product (GDP) (PPP) amounts to 5.71e+11, with China recording the highest GDP and Denmark having the lowest GDP. The mean years of schooling (MYS) is 7.9383 years, with Germany showcasing the highest MYS and Burkina Faso having the lowest MYS. The average urbanization rate (UR) stands at 57.048, with Singapore displaying the highest UR and Papua New Guinea having the lowest UR. Lastly, the mean CO2 emission per ton is 186,963.3, with China representing the highest CO2 emission and Sao Tome having the lowest CO2 emission [24].

**Table 2:** Descriptive statistics and IPS panel data unit root test results.

Variables	Mean	Std. Dev.	Min	Max	IPS P-value
LE (Years)	70.0689	9.213338	39.441	84.61561	0.000
IMR (Per 100,000)	27.25392	25.82926	1.5	138.1	0.000
IQ	−0.0299	0.8812	−1.9915	1.9611	0.000
CC	−0.0392	1.0037	−1.8158	2.4699	0.000
VA	0.0442	0.9443	−2.2333	1.8009	0.000
PS	−0.1084	0.9301	−3.1808	1.7601	0.000
RL	−0.0419	0.9714	−2.0085	2.1297	0.000
RQ	0.04375	0.9261	−2.6255	2.2605	0.000
GE	0.0173	0.9695	−2.2701	2.4370	0.000
HE (%GDP)	6.1327	2.5233	1.2636	20.4134	0.000
GDP (PPP)	5.71e+11	1.88e+12	2.77e+08	2.43e+13	0.000
MYS (Years)	7.9383	3.2448	1.1000	14.1517	0.000
UR (%TP)	57.0483	22.7857	8.2460	100	0.000
CO_2_ (Ton)	186963.3	808547.2	50	1.07e+07	0.000

Furthermore, the results of the IPS panel data unit root test reveal that all the variables included in the analysis are stationary, indicating the absence of spurious regression (refer to Table 2).

Table 3 presents the associations between institutional quality indices and the Infant Mortality Rate (LIMR) using the Fixed Effect Model. The results indicate that IQ, CC, VA, PS, RL, RQ, GE, LGDP, HE, MYS, and UR have a statistically significant negative effect on LIMR, while LCO2 has a significant positive effect. In other words, higher levels of institutional quality indices and other control variables are associated with lower infant mortality rates. Subgroup analysis (detailed in Appendix Table A1) was conducted to examine the relationship in high-income and low-income countries. In the high-income group, IQ, CC, VA, PS, RL, RQ, GE, LGDP, HE, MYS, and UR all demonstrate a significant negative effect on LIMR, while LCO2 shows a positive effect. Conversely, in low-income countries, IQ, GE, LGDP, HE, MYS, and UR have a significant negative effect on LIMR, CC and LCO2 have a significant positive effect, and VA, RL, PS, and RQ do not exhibit a significant relationship with LIMR.

**Table 3:** The estimation result of Fixed Effect for IQ and sub-indices: LIMR is depended variable (Eq (1)).

Variables	(1)	(2)	(3)	(4)	(5)	(6)	(7)
Constant	13.14792**	13.2815**	13.5968**	13.4772**	13.0995**	13.2313**	13.0837**
IQ	−0.1044**	⁎	⁎	⁎	⁎	⁎	⁎
CC	⁎	−0.0782**	⁎	⁎	⁎	⁎	⁎
VA	⁎	⁎	−0.0106**	⁎	⁎	⁎	⁎
PS	⁎	⁎	⁎	−0.0189**	⁎	⁎	⁎
RL	⁎	⁎	⁎	⁎	−0.0865**	⁎	⁎
RQ	⁎	⁎	⁎	⁎	⁎	−0.0404**	⁎
GE	⁎	⁎	⁎	⁎	⁎	⁎	−0.1190**
HE	−0.0106**	−0.0109**	−0.0101**	−0.0105**	−0.0102**	−0.0108**	−0.0113**
LGDP	−0.3846**	−0.3905**	−0.4041**	−0.3983**	−0.3823**	−0.3867**	−0.3813**
MYS	−0.0998**	−0.0992**	−0.0938**	−0.09573**	−0.0999**	−0.0989**	−0.0999**
UR	−0.0101**	−0.0096**	−0.0094**	−0.0097**	−0.0097**	−0.0101**	−0.0101**
LCO_2_	0.0756**	0.0740**	0.0705**	0.0718**	0.0727**	0.0724**	0.0747**
The first hypothesis was confirmed							

*, **, and *** Denote significance level at 1%, 5% and 10%, respectively.

The results from the system GMM estimation show that IQ, CC, VA, PS, RL, RQ, GE, LGDP, HE, MYS, and UR have a positive and significant effect on life expectancy (LE), indicating that higher levels of institutional quality indices and other control variables are associated with increased life expectancy. Conversely, LCO2 has a negative and significant effect, implying that higher levels of CO2 emissions are associated with lower life expectancy. Interestingly, the lagged variable LEit-1 demonstrates a stronger and more profound explanatory association with LE compared to other variables. This finding supports the main assumption that a higher level of life expectancy in previous years contributes to a higher level of life expectancy in the current year, highlighting the importance of considering the lagged effect of life expectancy in analyzing its determinants.

Subgroup analysis was conducted to examine the relationship within high-income and low-income groups, and the results are presented in Appendix Table A2. In the high-income group, IQ, CC, VA, PS, RL, RQ, GE, LGDP, HE, and MYS demonstrate a significant positive effect on life expectancy (LE). This suggests that higher levels of institutional quality indices, control of corruption, voice and accountability, political stability, rule of law, regulatory quality, government effectiveness, gross domestic product, total health expenditure, and mean years of schooling are associated with increased life expectancy. Conversely, LCO2 and UR have a negative and significant effect on LE in this group. On the other hand, in the low-income group, the results show that IQ, RL, RQ, and GE exhibit a negative effect on LE, although this relationship is not statistically significant. In contrast, VA, CC, PS, HE, MYS, UR, LCO2, and LGDP have a positive and significant effect on LE in this group. This implies that in low-income countries, variables such as voice and accountability, control of corruption, political stability, total health expenditure, mean years of schooling, urbanization rate, CO2 emissions, and gross domestic product have a positive impact on life expectancy. Overall, the subgroup analysis highlights the different patterns and factors influencing life expectancy in high-income and low-income countries.

The validity of the instrumental variables used in the estimations was assessed using the Sargan test. The results of the Sargan test showed that the p-values of the Sargan statistic for the system GMM regression were greater than five percent. Therefore, we did not reject the null hypothesis, indicating that the instrumental variables used in the estimations were valid. Additionally, the Arellano-Bond AR (1) and AR (2) autocorrelation tests were conducted on the residuals.

Furthermore, the robust test results of the Arellano-Bond test for serial correlation, specifically the first-order autocorrelation (AR-1) and the second-order autocorrelation (AR-2), indicate that the data supports the presence of serial autocorrelation. The test rejects the null hypothesis of second-order differenced at a statistical confidence interval of 0.05%.

The system GMM, Sargan, and Arellano-Bond test results are summarized in Table 4. Additional results of the F-Limer, Breusch-Pagan, and Haussmann tests can be found in Appendix Table A3. These tests provide important diagnostic information to ensure the reliability and robustness of the estimation results.

**Table 4:** The estimation results of system GMM for IQ and sub-indices: LE is depended variable (Eq (2)). with Sargan and Abond test results.

Variables		(1)	(2)	(3)	(4)	(5)	(6)	(7)
Constant		4.1376**	4.5743**	4.3211**	4.5160**	4.1364**	4.0968**	4.1261**
LE_it-1_		0.9183**	0.9156**	0.9165**	0.9166**	0.9161**	0.9188**	0.9175**
IQ		0.1430**	⁎	⁎	⁎	⁎	⁎	⁎
CC		⁎	−0.0203**	⁎	⁎	⁎	⁎	⁎
VA		⁎	⁎	0.0598**	⁎	⁎	⁎	⁎
PS		⁎	⁎	⁎	0.0251**	⁎	⁎	⁎
RL		⁎	⁎	⁎	⁎	0.1562**	⁎	⁎
RQ		⁎	⁎	⁎	⁎	⁎	0.1699**	⁎
GE		⁎	⁎	⁎	⁎	⁎	⁎	0.1209**
HE		0.04132**	0.0447**	0.0417**	0.0446**	0.0407**	0.0427**	0.0425**
LGDP		0.0716**	0.0373**	0.0554**	0.0423**	0.0761**	0.0771**	0.0714**
MYS		0.0564**	0.0624**	0.0609**	0.0617**	0.0578**	0.0543**	0.0592**
UR		0.0122**	0.0162**	0.0143**	0.0152**	0.0116**	0.0106**	0.0117**
LCO_2_		−0.1322**	−0.0999**	−0.1135**	−0.1083**	−0.1253**	−0.1371**	−0.1268**
Sargan (p-value)		0.9890	0.9967	0.9918	0.9964	0.9820	0.9834	0.9839
Abond	AR (1)	0.0000**	0.0000**	0.0000**	0.0000**	0.0000**	0.0000**	0.0000**
	AR (2)	0.0414**	0.0418**	0.0428**	0.0417**	0.0403**	0.0360**	0.0460**
The second hypothesis was confirmed								

*, **, and *** Denote significance level at 1%, 5% and 10%, respectively.

## 5 Discussion

In our study, we investigated the influence of institutional quality, along with its sub-indices, on two significant health indicators: life expectancy and infant mortality rates. To accomplish this, we first constructed a composite index (IQ) by combining six sub-indices that collectively reflect the overall quality of institutions. We then proceeded to estimate the impact of IQ on life expectancy and the infant mortality rate. Additionally, we employed dynamic panel data models to analyze the individual effects of each sub-index on these health outcomes. Furthermore, we conducted a separate analysis based on income groups to re-estimate all the models and assess any variations. Through this comprehensive approach, we aimed to gain insights into the relationship between institutional quality and health system outcomes, taking into account both the overall composite index and its individual components.

The findings depicted in Fig. 3 reveal that there is less variability in life expectancy among the indicators of institutional quality across different countries. Additionally, as we progress from lower institutional quality (negative part) to higher institutional quality (positive part), there is a clear improvement in life expectancy, indicating an increasing linear relationship. The results from Fig. 4 demonstrate that there is greater variability in infant mortality rates across different countries and among different institutional quality indicators. However, as institutional quality improves, moving from the lower end (negative part) to the higher end (positive part), there is a significant decrease in infant mortality with an increasing slope. This suggests a decreasing non-linear relationship between infant mortality and institutional quality indicators. In simpler terms, the impact of enhancing institutional quality on infant mortality is more pronounced compared to its effect on life expectancy. Even a slight improvement in a country's institutional quality leads to a notable reduction in infant mortality rates.

Another critical aspect of this Figure (Fig. 3) is the correlation between corruption control and life expectancy. The results from the figure indicated a positive association between improved corruption control and increased life expectancy. However, the GMM model yielded contrary findings, showing a negative relationship. In other words, when estimating the link between corruption control and life expectancy with a dynamic approach, accounting for the presence of an endogenous relationship, the harmful impact of the negative aspect of corruption control was revealed. This finding underscores the complexity of the relationship between these two indicators, which cannot be fully explained by simplistic models.

Our study findings support the notion that countries with stronger institutional quality tend to have lower infant mortality rates and higher life expectancy. These results are consistent with the research conducted by Rosenberg et al. (2018), who observed a correlation between improved political economy and reduced infant mortality rates [2]. Furthermore, Rahman et al. (2022) highlighted the role of good governance in promoting higher life expectancy specifically in ANZUS-BENELUX countries [10]. These previous studies provide additional support to our findings and further emphasize the importance of institutional quality in influencing health outcomes.

Enhanced institutional quality plays a vital role in ensuring improved healthcare services for various population groups, including pregnant mothers, children, and the elderly. It facilitates better access to healthcare professionals like physicians, nurses, and midwives, as well as essential medications and medical facilities. Consequently, it contributes to the establishment of an efficient, effective, and equitable health system [10,19,25].

Notably, the impact of institutional quality on life expectancy and infant mortality rates is more pronounced in high-income countries where development is more advanced and institutional frameworks hold greater significance. In these countries, the positive influence of institutional quality is evident due to the higher level of resources and emphasis placed on healthcare provision. On the other hand, in low-income countries, various factors such as underdevelopment, socioeconomic challenges, civil unrest, ethnic disparities, and weak democratic governance have contributed to a diminished focus on institutional quality and its performance. Consequently, improving institutional quality emerges as a critical pathway for enhancing health outcomes in both high and low-income countries [14,26].

The analysis of sub-indices revealed that the Control of Corruption has a significant and positive effect on increasing life expectancy and reducing infant mortality rates. This finding is consistent with a study conducted by M.V. Achim et al. (2020), which demonstrated that higher levels of corruption are associated with a decline in physical and mental health, while better control of corruption leads to improvements in these areas [13].

In the context of the healthcare system, effective control of corruption entails implementing measures to manage the provision of informal and inefficient services, ensuring fair access to neonatal and maternal healthcare, allocating budgets optimally for primary and preventive services, and addressing issues of information asymmetry, among other actions. Countries with stronger anti-corruption policies and mechanisms tend to experience lower infant mortality rates [9,11,27].

By promoting transparency, accountability, and integrity in healthcare service delivery, efforts to control corruption can lead to improved health outcomes. These measures foster public trust in the healthcare system, facilitate the equitable distribution of resources, and encourage the efficient use of healthcare resources, ultimately benefiting maternal and child health.

Subgroup analysis revealed divergent results regarding the impact of control of corruption on health outcomes. Specifically, while corruption control demonstrated a positive association with health outcomes in high-income countries, it displayed a negative association in low-income countries. It is crucial to note that corruption control encompasses two distinct dimensions.

In the positive dimension, effective control of corruption indicates good governance practices and institutions that promote transparency, accountability, and integrity. In high-income countries with stronger governance mechanisms, the control of corruption is associated with improved health outcomes, including lower infant mortality rates. This suggests that when corruption is effectively managed, resources are allocated efficiently, healthcare services are delivered more equitably, and public trust in the healthcare system is fostered [13,28,29].

However, in the negative dimension, stricter corruption control may reflect a higher prevalence of corruption within a society. In low-income countries where corruption is more pervasive, efforts to control corruption may be less successful, and public trust in the government and healthcare system may be eroded. This can result in a decline in life expectancy and compromised health outcomes. Factors such as limited resources, weak governance structures, and societal challenges contribute to the complex relationship between corruption control and health outcomes in low-income countries [13,28,29].

Overall, the contrasting results highlight the importance of considering contextual factors when examining the impact of control of corruption on health outcomes. Effective corruption control, accompanied by strong governance practices, is a critical aspect of promoting positive health outcomes, particularly in high-income countries. In low-income countries, addressing corruption and strengthening governance structures are essential steps towards improving health outcomes and fostering public trust in the healthcare system.

Previous studies have emphasized the significance of transparency enhancement as a key policy in reducing corruption. Enhancing transparency involves implementing various initiatives such as improving digital infrastructure, strengthening the role of non-governmental organizations (NGOs), and promoting the free flow of information [11,27,30].

In high-income countries, where governance mechanisms are generally more advanced, improving corruption control has demonstrated positive effects on health outcomes, particularly in terms of life expectancy. The presence of strong governance structures and anti-corruption measures facilitates the efficient allocation of resources, equitable access to healthcare services, and the establishment of public trust in the healthcare system [9,11,28].

Conversely, in low-income countries, corruption is often perceived as a prevalent and deeply rooted issue, and there may be limited incentives or resources to effectively combat it. The context of low-income countries differs significantly from high-income countries, and as such, corruption control policies cannot be uniformly applied. To effectively address corruption in low-income countries, it is crucial to develop tailored policies that take into account the specific political, economic, and social contexts in which corruption operates [9,28,30].

Recognizing these contextual differences, efforts to tackle corruption in low-income countries should encompass strategies that address underlying causes, such as poverty, inequality, and weak institutions. This may involve capacity-building initiatives, strengthening anti-corruption agencies, promoting civic engagement and participation, and fostering international collaborations to provide technical assistance and support [9,28,30].

In summary, while enhancing transparency and improving corruption control are vital in combating corruption across all countries, the approaches and strategies employed should be context-specific. Tailoring corruption control policies to the specific challenges and circumstances of low-income countries is essential for effectively addressing corruption and promoting positive health outcomes in those settings.

Based on our findings, the development of voice and accountability is strongly associated with a reduction in infant mortality rates (LIMR) and an improvement in life expectancy. A robust voice and accountability framework encompass key elements such as the presence of democracy and its respect, mechanisms for public monitoring, freedom of the press and social media, and the recognition of the right to voice and accountability. In societies with established democratic processes, the monitoring of the health system's performance is more accurate, and the system itself is held accountable [11,31]. Consequently, fostering accountability, which includes upholding democratic principles, enhances the overall performance of the health system, leading to a decrease in LIMR and an improvement in life expectancy [9,12].

Furthermore, our findings indicate that an improvement in political stability is associated with a decrease in LIMR and an improvement in life expectancy. Enhanced political stability encompasses elements such as economic, social, and political predictability, the presence of a developed and reliable judicial system, the availability of legal recourse against the government, and efforts to combat terrorism [32]. A stable political environment fosters public trust, which, in turn, strengthens government authority. This government authority contributes to the stability of the economic system and ultimately ensures the provision of efficient, effective, and equitable healthcare services [10,26,33,34].

Taken together, our findings highlight the importance of promoting voice and accountability as well as political stability for improving health outcomes. By establishing democratic processes, encouraging public monitoring, upholding freedom of the press, and ensuring political stability, societies can create an environment that facilitates the provision of high-quality healthcare services. These factors ultimately contribute to the reduction of infant mortality rates and the improvement of life expectancy.

Improvements in regulatory quality and the rule of law have been found to be associated with a reduction in the Infant mortality rate (LIMR), with a more significant impact on life expectancy. Regulatory quality in the healthcare system encompasses factors such as the presence of healthcare guidelines, laws safeguarding patients and pregnant mothers, social health insurance and retirement regulations, mechanisms for public monitoring, and the establishment of competition rules, among others. The rule of law in healthcare systems entails attentiveness to and compliance with these regulations [14,35,36].

Specifically, in the high-income group of countries, enhancing voice and accountability, political stability, the rule of law, and regulatory quality contribute to improved health outcomes. On the other hand, in the low-income group, the rule of law emerges as the primary influential factor. In countries grappling with wars, coups, poverty, and natural disasters such as droughts and earthquakes, the primary need is for the establishment of the rule of law to foster political, social, and economic stability, with comparatively less emphasis on other factors.

The government plays a critical role in the healthcare system, and its effectiveness is crucial for ensuring public health [3]. Consequently, an increase in government effectiveness leads to a decrease in the infant mortality rate (LIMR) and an improvement in life expectancy. Effective government in healthcare involves implementing performance-based budgeting, allocating resources based on cost-effectiveness, developing healthcare guidelines, establishing government health financing and social health insurance systems, providing primary health services such as prevention and screening, and streamlining bureaucratic procedures through monitoring and fundamental reforms, among other public goods [[37], [38], [39]].

The World Health Organization's 2022 health report highlights the central position of governments in addressing health issues, emphasizing the significance of enhancing government effectiveness. Therefore, prioritizing the improvement of government effectiveness is of utmost importance as it plays a key role in enhancing life expectancy and reducing the infant mortality rate [4]. Enhancing government effectiveness encompasses factors such as garnering popular support, implementing a meritocracy system, and fostering improvements in public trust [40,41].

According to the World Economic Forum's (WEF) Global Risk Report 2023, climate change is identified as the primary concern for the future of our world [42]. To evaluate the impact of CO2 emissions on life expectancy and the burden of climate change-related diseases, we examined the relationship between CO2 emissions from fossil fuel consumption and the Infant mortality rate (LIMR). Our findings reveal that increased CO2 emissions have a positive effect on LIMR and a negative effect on life expectancy. Therefore, it is imperative to intensify efforts to reduce CO2 emissions and mitigate their impact on public health [41].

Furthermore, research by E-Z Wang and M. Yang (2022) demonstrates that enhancing the quality of institutions contributes to the reduction of CO2 emissions [43]. Our own study also indicates that improving institutional quality leads to better health outcomes. Thus, enhancing institutional quality emerges as a significant approach to addressing the health consequences of climate change. Additionally, countries with higher GDP, effective healthcare systems, improved education, and higher urbanization rates have shown better health outcomes in previous years [10,33]. Particularly, in low-income countries, improving national income, developing the education system, enhancing infrastructure, and expanding access to primary healthcare services have contributed to improved health outcomes. Similarly, countries with higher life expectancy in the past have exhibited better health outcomes, as evidenced by a positive and statistically significant lagged effect on life expectancy.

## 6 Limitations, strengths and conclusion

Like other studies, our study had some limitations. One of the important limitations is that this study is an ecological study. Therefore, generalizing its results to a country is not correct. To answer the research question in a single country, another study should be conducted at the level of that country. Another limitation is related to the potential bias or error in the measurement of the data used.

Despite the limitations, our study had several strengths. High sample size (N = 3160), global scale (inclusion of 158 countries in the study), examination of all dimensions of institutional quality including control of corruption, voice and accountability, political stability, rule of law, regulatory quality, and government effectiveness, and estimating the models separately for two groups of high and low income countries were among the most important strengths of this study.

The findings of this study can help global policymakers in planning to change the institutional quality and its sub-indices towards better health outcomes. To be more precise, the findings of this study can serve as solid evidence for health policymakers to support better advocacy efforts.

Finally, the results of the models have confirmed the two fundamental hypotheses of this study. It can be confidently asserted that institutional quality plays a decisive role in the health systems of countries, and its improvement over the last 20 years has directly contributed to increased life expectancy and reduced infant mortality rates. Our research has unveiled the significant impact of institutional quality on the overall performance of healthcare systems. Specifically, enhancing institutional quality is associated with upholding democratic and meritocratic principles, developing infrastructure, improving tax and subsidy systems, allocating budgets optimally, advancing public education, and increasing accessibility to primary healthcare services. Moreover, the influence of institutional quality is more pronounced in the high-income group compared to the low-income group. This highlights the importance of institutions in countries with stable economic, political, social, and governmental conditions, whereas nations facing conflicts, natural disasters, and disease outbreaks encounter additional challenges. Consequently, in high-income countries, improving institutional quality leads to better health outcomes. However, in low-income countries, the focus should be on increasing national income, developing the education system, improving infrastructure, and enhancing access to primary healthcare services in order to achieve improved health outcomes. Therefore, it is crucial to acknowledge that policies successful in high-income countries may not directly apply or be effective in low-income countries due to their distinct contexts and needs.

## 7 Policy implication

The findings of this study can help global policymakers in planning to change the institutional quality and its sub-indices towards better health outcomes. To be more precise, the findings of this study can serve as solid evidence for health policymakers to support better advocacy efforts.

## Authors' contributions statement

Alireza Hadipour: Wrote the paper, Conceived and designed the experiments, Performed the experiments, Analyzed, and interpreted the data, Contributed reagents, materials, analysis tools or data. Mohsen Bayati: Wrote the paper, contributed reagents, materials, analysis tools, Performed the experiments.Sajad Delavari: Wrote the paper, Performed the experiments.

## Funding

This paper was financially supported by 10.13039/501100004320Shiraz University of Medical Sciences with grant number 26558 The funder had no role in the study design, data collection, analysis, and interpretation, and writing of the manuscript.

## Ethics approval and consent to participate

The project was found to be in accordance to the ethical principle and national norms and standards for conducting medical research. The study protocol was approved by the ethics committee of Shiraz University of Medical Sciences under code IR. SUMS. NUMIMG.1401.067.

## Data availability

The data used in this study are publicly and available in.

The World Bank (https://info.worldbank.org/governance/wgi/).

WHO (https://www.who.int/data/gho/publications/world-health-statistics).

UN (https://unstats.un.org/unsd/demographic-social/)

## Declaration of competing interest

The authors declare that they have no known competing financial interests or personal relationships that could have appeared to influence the work reported in this paper.

## Figures and Tables

**Table A.1:** The estimation result of FE for IQ and sub-indices by income group analysis: LIMR is depended variable (Eq (1))

Variables		(1)	(2)	(3)	(4)	(5)	(6)	(7)
Constant	HI	13.6163	13.7760	14.1733	14.0758	12.5031	12.3478	12.038
	LI	12.1612	12.4118	12.5031	12.2707	13.4296	13.6221	14.0758
IQ	HI	−.1507**	⁎	⁎	⁎	⁎	⁎	⁎
	LI	−.0379**	⁎	⁎	⁎	⁎	⁎	⁎
CC	HI	⁎	−.1384**	⁎	⁎	⁎	⁎	⁎
	LI	⁎	.0315**	⁎	⁎	⁎	⁎	⁎
VA	HI	⁎	⁎	−.0442**	⁎	⁎	⁎	⁎
	LI	⁎	⁎	.0157	⁎	⁎	⁎	⁎
PS	HI	⁎	⁎	⁎	−.0242**	⁎	⁎	⁎
	LI	⁎	⁎	⁎	−.0387	⁎	⁎	⁎
RL	HI	⁎	⁎	⁎	⁎	.01573**	⁎	⁎
	LI	⁎	⁎	⁎	⁎	−.1594	⁎	⁎
RQ	HI	⁎	⁎	⁎	⁎	⁎	.0152**	⁎
	LI	⁎	⁎	⁎	⁎	⁎	−.0632	⁎
GE	HI	⁎	⁎	⁎	⁎	⁎	⁎	−.0593**
	LI	⁎	⁎	⁎	⁎	⁎	⁎	−.0242**
HE	HI	−.0218**	−.0223**	−.0211**	−.0216**	−.0029**	−.0027**	−.0034**
	LI	−.0028**	−.0025**	−.0029**	−.0029**	−.0202**	−.0223**	−.0216**
LGDP	HI	−.4437**	−.4509**	−.4665**	−.4628**	−.3329**	−.3264**	−.3126**
	LI	−.3187**	−.3294**	−.3329**	−.3236**	−.4366**	−.4418**	−.4628**
MYS	HI	−.0758**	−.0770**	−.0688**	−.0691**	−.1309**	−.1339**	−.1355**
	LI	−.1341**	−.1342**	−.1309**	−.1313**	−.0758**	−.0746**	−.0691**
UR	HI	−.0065**	−.0058**	−.0059**	−.0056**	−.0107**	−.0108**	−.0117**
	LI	−.0111**	−.0107**	−.0107**	−.0114**	−.0060**	−.0065**	−.0056**
LCO_2_	HI	.1258**	.12,478**	.1127**	.1094**	.0522**	.0543**	.0503**
	LI	.0516**	.0562**	.0523**	.0522**	.1224**	.1171**	.10,941**

*, **, and *** Denote significance level at 1%, 5% and 10%, respectively.

**Table A.2:** The estimation results of system GMM for IQ and sub-indices by income-group analysis: LE is depended variable. (Eq (2))

Variables		(1)	(2)	(3)	(4)	(5)	(6)	(7)
Constant	HI	3.8539	4.1612	3.9352	3.9792	3.8671	3.9182	3.9640
	LI	1.8632	1.5790	2.2081	2.1516	1.6280	2.1572	1.9667
LE_it-1_	HI	.9330**	.9378**	.9346**	.9367**	.9320**	.9316**	.9312**
	LI	.9442**	.9437**	.9438**	.9446**	.9463**	.9451**	.9439**
IQ	HI	.1150**	⁎	⁎	⁎	⁎	⁎	⁎
	LI	−.0514	⁎	⁎	⁎	⁎	⁎	⁎
CC	HI	⁎	−.0412**	⁎	⁎	⁎	⁎	⁎
	LI	⁎	−.1309**	⁎	⁎	⁎	⁎	⁎
VA	HI	⁎	⁎	.0440**	⁎	⁎	⁎	⁎
	LI	⁎	⁎	.0570**	⁎	⁎	⁎	⁎
PS	HI	⁎	⁎	⁎	.0436**	⁎	⁎	⁎
	LI	⁎	⁎	⁎	.0133**	⁎	⁎	⁎
RL	HI	⁎	⁎	⁎	⁎	.1251**	⁎	⁎
	LI	⁎	⁎	⁎	⁎	−.1169	⁎	⁎
RQ	HI	⁎	⁎	⁎	⁎	⁎	.1519**	⁎
	LI	⁎	⁎	⁎	⁎	⁎	−.0416	⁎
GE	HI	⁎	⁎	⁎	⁎	⁎	⁎	.1298**
	LI	⁎	⁎	⁎	⁎	⁎	⁎	−.0733
HE	HI	.0378**	.0545**	.04341**	.0478**	.0356**	.0343**	.0368**
	LI	.0487**	.0480**	.0482**	.0132**	.0475**	.0480**	.0472**
LGDP	HI	.0913**	.0477**	.0759**	.0658**	.0949**	.0957**	.0934**
	LI	.1919**	.2037**	.1806**	.0488**	.1975**	.1751**	.1836**
MYS	HI	−.0381	−.0410	−.0375	−.0409	−.0373	−.0338	−.0342
	LI	.2312**	.2358**	.2225**	.2240**	.2225**	.2291**	.2247**
UR	HI	−.0035**	−.0041**	−.0033**	−.0038**	−.0038**	−.0039**	−.0036**
	LI	−.0096**	−.0099**	−.0086**	−.0091**	−.0099**	−.0095**	−.0088**
LCO_2_	HI	−.0585**	−.0138**	−.0427**	−.0324**	−.0596**	−.0673**	−.0649**
	LI	−.42,981**	−.4344**	−.4258**	−.4235**	−.4311**	−.4220**	−.4187**

*, **, and *** Denote significance level at 1%, 5% and 10%, respectively.

**Table A.3:** The F-Limer, breusch-pagan and Hussmann test results

Tests	variables	Statistic	P-Value
F-Limer	LIMR	F (157, 2996) = 323.24	0.000
	LE	F (157, 2996) = 303.35	0.000
breusch-pagan	LIMR	Chibar2: 23566.79	0.000
	LE	Chibar2: 20329.34	0.000
Hussmann	LIMR	Chi2 = 493.23	0.000
	LE	Chi2 = 321.86	0.000

According to tests result we choose panel and fixed effect model as general model but for Life expectancy -based on fundamental assumption and previous literature-we choose System GMM.
